# Clinical Diagnostics of Bacterial Infections and Their Resistance to Antibiotics—Current State and Whole Genome Sequencing Implementation Perspectives

**DOI:** 10.3390/antibiotics12040781

**Published:** 2023-04-19

**Authors:** Ekaterina Avershina, Abdolrahman Khezri, Rafi Ahmad

**Affiliations:** 1 Department of Biotechnology, Inland Norway University of Applied Sciences, Holsetgata, 222317 Hamar, Norway; 2 Institute of Clinical Medicine, Faculty of Health Science, UiT The Arctic University of Norway, Hansine Hansens veg, 189019 Tromsø, Norway

**Keywords:** antimicrobial resistance, rapid diagnostics, whole genome sequencing, microbial genomics, bacterial culture, machine learning, enabling technologies

## Abstract

Antimicrobial resistance (AMR), defined as the ability of microorganisms to withstand antimicrobial treatment, is responsible for millions of deaths annually. The rapid spread of AMR across continents warrants systematic changes in healthcare routines and protocols. One of the fundamental issues with AMR spread is the lack of rapid diagnostic tools for pathogen identification and AMR detection. Resistance profile identification often depends on pathogen culturing and thus may last up to several days. This contributes to the misuse of antibiotics for viral infection, the use of inappropriate antibiotics, the overuse of broad-spectrum antibiotics, or delayed infection treatment. Current DNA sequencing technologies offer the potential to develop rapid infection and AMR diagnostic tools that can provide information in a few hours rather than days. However, these techniques commonly require advanced bioinformatics knowledge and, at present, are not suited for routine lab use. In this review, we give an overview of the AMR burden on healthcare, describe current pathogen identification and AMR screening methods, and provide perspectives on how DNA sequencing may be used for rapid diagnostics. Additionally, we discuss the common steps used for DNA data analysis, currently available pipelines, and tools for analysis. Direct, culture-independent sequencing has the potential to complement current culture-based methods in routine clinical settings. However, there is a need for a minimum set of standards in terms of evaluating the results generated. Additionally, we discuss the use of machine learning algorithms regarding pathogen phenotype detection (resistance/susceptibility to an antibiotic).

## 1. Introduction

Antibiotics are natural or synthetic chemical compounds that inhibit bacterial growth and are the first-line drugs against bacterial infection. Bacteria have rapidly developed and spread antibiotic resistance across different bacterial classes in response to unnecessary antibiotic use, flaws in proper diagnostic tools, poor hygiene conditions, and suboptimal healthcare practices. During resistance development, antibiotics gradually become less effective, and bacteria can adapt and continue to grow in their presence [1].

Antibiotic resistance arises mainly via four different mechanisms: reducing efflux transport, target modification, limiting drug uptake, and enzyme-catalyzed inactivation [2,3]. Efflux pumps are a large family of protein pumps in bacteria that emit antibiotics from inside the cell to the outside [4]. Bacteria also develop resistance toward a specific class of antibiotics upon a series of DNA mutations or producing specific enzymes, resulting in modification of the targets of that class of antibiotic [5]. Alternatively, some proteins can bind to antibiotics or their targets, reducing antibiotic uptake [6]. Bacteria also inactivate antibiotics by producing enzymes that recognize and destroy antibiotics’ structure [7]. It has been shown that bacteria develop resistance via post-translational mechanisms as well [8]. These resistance mechanisms can be categorized as intrinsic or expected resistance (found across all strains/bacteria) or acquired (first appearing only in a few strains and then spreading to microorganisms of distant taxonomical relatedness). Therefore, acquired resistance poses a greater risk to human and animal health.

Antibiotic resistance, although having existed before antibiotics were discovered [9], is strongly driven via antibiotic use in human and veterinary medicine. Since the 1960s, antibiotic resistance has dramatically increased, fast becoming a global public health concern [10,11]. A recent meta-analysis of resistant bacteria burden on human health and well-being revealed that in 2019 alone, a striking 1.27 M deaths were caused directly by antibiotic-resistant bacteria (ARBs), and 4.95 M deaths were associated with ARBs. This number has surpassed the human immunodeficiency virus (HIV) and malaria [11]. Given the elevated use of antibiotics in 2020 due to the coronavirus disease (COVID-19) pandemic and low AMR resilience in many countries, this number has potentially increased in recent years. Recently published data indicate that the mortality rate increased in COVID patients who received antibiotics [12,13]. A recent study based on metagenomic sequencing of 757 sewage samples from 243 cities in 101 countries suggested that certain geographies are more prone to transmission events and should receive additional attention [14]. An estimated death rate of more than 10 million people annually by 2050 may be realistic due to antibiotic resistance [15,16,17]. From a healthcare perspective, antibiotic resistance causes more extended hospitalization of patients suffering from an infection. This eventually imposes a tremendous additional cost for the hospitals and could cost an extra USD 300 billion to more than USD 1 trillion annually by 2050 globally [18].

Furthermore, antibiotic resistance can indirectly affect the success rate of other procedures, such as surgeries and chemotherapy [19,20]. From an economic perspective, antibiotic resistance has multiple effects on the global economy. For instance, it has been reported that antibiotic resistance has led to a shortage in protein resources (sick animals not responding to antibiotic treatment and/or antibiotic residues in meat/milk precluding their consumption) [21], a pronounced increase in poverty and inequity [22], deficiency in productivity due to sickness [23], and significantly decreased global trade [24].

## 2. Current Methods for the Clinical Diagnosis of Bacterial Infection

### 2.1. Pathogen Identification

#### 2.1.1. Culture-Dependent Techniques

In these methods, culturable bacteria are enriched using a non-specific or selective medium and then characterized based on their morphology and metabolic traits. [25] Biochemical tests are based on qualitative biochemical reactions, e.g., visible changes in media because of bacterial metabolic activity. In colony morphology, microorganisms are identified based on the morphological properties of a specific strain [25]. The conventional approaches benefit from simplicity, reproducibility, and low cost. However, one should remember that the enrichment of microorganisms is a labor-intensive and time-consuming procedure, and colonies often require 1–3 days and sometimes longer to grow [26]. Besides that, correct pathogen identification based on culture often requires vast expertise, which might lead to inaccurate diagnosis [27]. To minimize such inaccuracy in biochemical methods, analytical profile index (API) tests [28] and automated systems such as VITEK (bioMerieux, Inc., Marcy-l’Étoile, France) have been developed [29].

The principle of immunological techniques is based on the interaction between diagnostic antibodies and certain antigenic elements of bacteria. Among different immunological assays, the enzyme-linked immunosorbent assay (ELISA) is the most frequently used technique in microbiology [30]. The ELISA technique became popular for its ability to identify multiple pathogens simultaneously, high-throughput capacity, low cost, and the opportunity to gauge the invading pathogens. Nonetheless, immunological techniques suffer from their dependency on the number of antibodies against pathogens epitopes (in the case of indirect ELISA). Furthermore, as some antigens are shared between bacterial species, the antigen–antibody binding is not specific. Therefore, immunological techniques lack selectivity and sensitivity.

#### 2.1.2. Mass Spectroscopy

Several mass spectroscopy (MS) techniques have been developed in recent years. Matrix-assisted laser desorption ionization-time of flight mass spectrometry (MALDI-ToF MS) is the most common MS technique to identify bacterial species and even strains [31]. In this technique, the prepared bacteria are exposed to short laser pulses, and consequently, their proteins become ionized. The ions travel through a vacuum tube and are registered by the sensor, thus creating a spectrum. The resulting spectrum is then compared with a database of known bacteria spectra. A more in-depth methodology of MALDI-ToF is described by Singhal et al. [32]. Although microbial identification using MALDI-ToF MS is fast and accurate, the protein spectra for closely related species, e.g., *Escherichia coli* and *Shigella*, are highly similar [33], which hampers correct identification.

Furthermore, initial and maintenance costs are the major concerns [32]. It has been estimated that a MALDI-ToF MS system requires EUR 200,000 per year for maintenance, limiting its usage to large hospitals capable of covering these costs [34]. Furthermore, the limited application of the MALDI-ToF MS system to the early detection of antibiotic resistance [35] makes this technique less attractive for the AMR field. However, a recent study demonstrated improved prediction of antibiotic resistance based on MALDI-ToF data by implementing a machine learning algorithm [36].

#### 2.1.3. Nucleic-Acid-Based Techniques

Nucleic-acid-based techniques, such as the polymerase chain reaction (PCR), reverse transcriptase PCR (RT-PCR), and transcription-mediated amplification (TMA), are used in microbial diagnostic laboratories. The main feature of such techniques is their ability to amplify specific regions of the pathogen genome (conserved genes or 16 S ribosomal RNA genes), commonly used as taxonomic markers [37,38]. Although nucleic-acid-based techniques are highly sensitive and accurate, bacterial infections are, in some cases, caused by multiple bacteria, which might cause species–species or strain–strain interactions in this type of assay [39]. The PCR also requires primer design, which limits flexibility as one needs to have prior knowledge of the bacterial species present in the clinical samples. Generally, PCR techniques are culture-independent; however, a low-grade infection may require pathogen enrichment before analysis [40]. To overcome such limitations, some syndromic panels (taking benefits from the multiplexed real-time PCR) have recently been developed and certified for diagnostics, which allows users to quickly identify the most common pathogens [41,42]. The main disadvantages of these panels are the limited number of pathogens and AMR resistance profiles covered by the currently available kits [41,42].

### 2.2. Antibiotic Susceptibility Profiling

#### 2.2.1. Phenotypic Techniques

The broth dilution test is one of the initial methods in antibiotic resistance screening. Here, a series of antibiotic dilutions within a liquid culture medium are prepared, and the bacteria of interest are inoculated. Then, the tubes are incubated overnight at 37 °C, and bacterial growth is detected. The lowest concentration of antibiotics that inhibit growth is considered the minimal inhibitory concentration (MIC) [43]. The method is highly reproducible, and the MIC value is highly informative. However, users might face time limitations and a lack of flexibility regarding antibiotic choice [44].

With the antimicrobial gradient method, bacteria are cultured on an agar medium and co-incubated with a few plastic test strips, each containing a gradient concentration of a specific antibiotic. The point where the lower segment of the ellipse-shaped growth inhibition zone touches the test strip would be considered the minimum inhibitory concentration (MIC) value [45]. The method is simple and easy to run and requires no specific expertise; however, like the broth dilution test, this method is limited to the number of antibiotics and could be costly if one tends to test multiple antibiotics [46,47].

Disc diffusion tests have been commonly used for years in many diagnostic laboratories. The single concentration of antibiotics impregnated into small round paper is placed on the agar surface with cultured bacteria. After 16–24 h incubation at 37 °C, the zone diameter with no bacterial growth close to each antibiotic disc is measured and compared with the reference values [43]. This method is simple and cheap to run, while to date, no automated system has been developed for fast screening of the no-growth zone on the plates.

The main drawback of phenotypic methods is that they take up to a few days due to culturing. However, the new culture-based technology, Accelerate PhenoSystem (APS, Accelerate Diagnostics, Inc., Tucson, AZ, USA), allows pathogen ID and MIC values to be obtained within 2 and 7 h, respectively. The system uses the fluorescence in situ hybridization (FISH) technique for identifying common pathogens in bloodstream infections and morphokinetic cellular analysis for AST [43,45,48]. Another recent technology that allows same-day pathogen ID and AST is based on acoustic-enhanced flow cytometry [49]. When used for infection identification in peritoneal-dialysis-associated peritonitis, this technology provided an infection confirmation within 1 h and AST within 3–6 h after sample reception.

#### 2.2.2. Molecular Techniques

The PCR and the qPCR (in some cases) are the most valuable techniques for quantifying and detecting resistance genes in bacteria in clinical laboratories [50]. New PCR variants such as digital droplet PCR (ddPCR) offered even higher sensitivity than qPCR for SARS-CoV-2 detection [51]. Another study suggested a hybrid combination of the PCR and the isothermal PCR as a highly sensitive method for SARS-CoV-2 diagnostics [52]. The advantage of the qPCR over the PCR is the quantitative measurement of the targeted gene(s). The main weakness of these methods is that one needs to decide about the targeted gene(s) in advance. Therefore, both assays have a limited capacity to detect unexpected/unanticipated genes.

Furthermore, the variants of resistance genes might be difficult to detect. Another critical point in the field of antibiotic resistance is the discrepancy between the nucleic acid amplification test and AST results. Culture-dependent AST results are considered the gold standard for resistance detection. The Clinical and Laboratory Standards Institute (CLSI) has provided specific guidelines for resolving such disagreements in Gram-positive organisms and ESBL and carbapenemase in Gram-negative organisms [53,54].

## 3. The Potential of Whole Genome Sequencing (WGS) for Use in Clinical Routine

### 3.1. DNA Sequencing Technologies

Sequencing technology has seen tremendous development during the last few decades. The human genome project was one of the main reasons for the rapid establishment of high-throughput WGS as a valuable tool for studying an organism’s genome. The first generation of sequencing was based on chain-terminating inhibitors proposed by Sanger et al. [55]. In Sanger sequencing, DNA fragments are PCR amplified, and then denaturized PCR products are further used as templates. The new DNA strand, complementary to the denaturized PCR product, is synthesized using chain-terminating fluorescence-labeled di deoxynucleotides triphosphates (ddNTPs). Whenever DNA polymerase incorporates a ddNTP, the extension terminates. Then, these newly synthesized strands are loaded into capillary electrophoresis. The time of their passage through the capillary depends on the size of the fragments—the shorter the fragment, the faster it passes. Upon exit from the capillary, a laser excites fluorescent tags of the ddNTPs in each fragment, recording the emitted light. This method was previously widely used for microbial species identification [56]. Sanger sequencing does not allow high throughput and is time- and resource-demanding.

The limitations of Sanger sequencing led to the development of the next (second)-generation sequencing (NGS) methods at the beginning of 2005 [57]. The main difference between Sanger sequencing and NGS is that NGS allows the sequencing of millions of fragments simultaneously. One of the most popular NGS technologies is Illumina sequencing (Illumina Ltd., San Diego, CA, USA). The high-throughput nature of NGS decreased the cost and time of pathogen identification significantly and has the potential to be considered as a routine diagnostic method [58]. For instance, NGS contributed significantly to diagnosing more than 150 genetic diseases [59]. Although NGS was an evolutionary step in sequencing technology, it also suffers from specific limitations, such as high initial costs and short read lengths (maximum 300 bp). Furthermore, complex bioinformatic tools and techniques are required to analyze data after sequencing.

Third-generation sequencing (TGS) emerged in early 2011 with the introduction of the PacBio system [57]. TGS can produce long reads with an average length of 10 kb and a maximum length of 80 kb [60,61], covering the repeating regions in the genome, which makes the assembly and characterization of large structural changes in the genome less challenging [57]. However, the application of the PacBio system in clinical microbiology is limited by its initial costs, portability, and the complexity of data analyses and software setup [62]. Another commonly used example of TGS was introduced by Oxford Nanopore Technology (ONT). Nanopore sequencing is becoming a popular sequencing method for several reasons.

First, considering the portability of some of its variants (MinION and Flongle), real-time sequencing, and data analyses, this technology greatly reduces the time required from sample preparation to results. Moreover, nanopore sequencing allows direct DNA sequencing to omit the PCR amplification step (depending on the library preparation kit); therefore, the sequencing data have a more consistent genome coverage [60]. Nanopore technology comprises nano-sensors and specific “channel“ types through which single-stranded DNA penetrates during the sequencing run. Passaging the DNA through the pore results in electric current changes sensed by reader proteins. The current change is base specific, and all alterations in the electrical signal can be translated into nucleotide bases. Despite nanopore sequencing’s significant usefulness, one of its main drawbacks is higher sequencing error compared to Illumina, which is now gradually improved by new chemistry, kits, and more accurate base calling algorithms [63,64]. In addition, the bioinformatic toolkits for nanopore sequencing data analysis need to be better developed compared to tools designed for NGS data analyses [65].

### 3.2. WGS Application in Clinical Settings

The currently implemented methods for pathogen identification and antibiotic resistance detection either require a long time (culturing-based techniques) or can only detect predefined targets (PCR-based techniques and phenotypic assays). Regarding culture-based methods, there is a risk of overlooking fastidious pathogens [66]. Moreover, testing AMR for antibiotic susceptibility testing (AST) can further take 18–24 h [67]. All these factors can potentially be eliminated using untargeted whole genome sequencing (WGS), which has become more affordable (although still expensive compared to conventional methods) and, importantly, portable. Although WGS cannot be widely implemented in routine clinical labs at the time of publication, there is a growing body of research on the potential of WGS in clinical routine infection profiling, especially in severe cases (f.ex. sepsis) where rapid and proper antibiotic treatment initiation is a matter of life or death. In addition, direct sequencing methods can also detect dead bacteria when antibiotic therapy has been administered before sampling [68].

WGS commonly refers to the sequencing genome of a single organism. Biological samples are rarely mono-colonized, however. When whole genomes of several species are sequenced simultaneously, it is referred to as metagenome sequencing (meta-WGS). Although not routine at the time of publication, meta-WGS paves its way to being widely used in several clinical applications. Illumina sequencing has a 99–99.9% average accuracy rate and is thus often regarded as the gold standard of sequencing [69]. ONT platforms, on the other hand, despite having higher error rates, are more suitable for clinical application since they output sequencing information in real-time. Real-time ONT metagenomic sequencing (mONS), for example, has been successfully implemented in lower respiratory tract infections (sputum), urinary tract infections (urine), central nervous system (cerebral spinal fluid), sepsis (blood), surgical site infections, and orthopedic devices with up to 100% sensitivity and specificity in pathogen detection (Table 1). These studies demonstrate that both bacterial identification and antibiotic resistance genes detection were reached within hours of sample acquisition. Considering that conventional bacterial identification and antibiotic susceptibility testing may take up to a week, mONS clearly offers a significant leap toward real-time diagnostics in the future.

To date, the use of meta-WGS in routine clinical diagnostics is limited by several factors, including complex workflows, still slow turnaround times, and relatively high costs. Moreover, there is the need for extensive data analysis, which requires both computational infrastructure and bioinformatics expertise. With sequencing prices constantly decreasing, data mining becomes a bottleneck for using meta-WGS in clinical routine.

### 3.3. WGS Data Analysis

**Figure 1:**
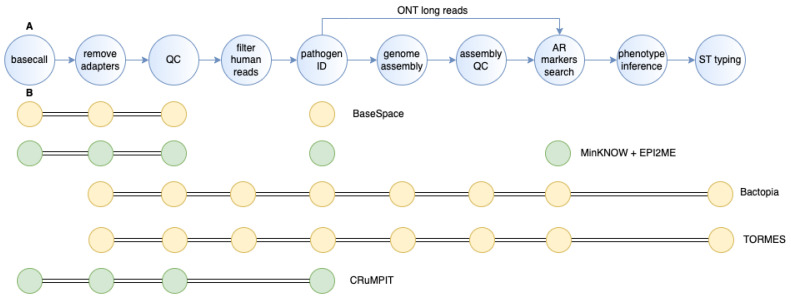
Pipelines for sequencing data analysis for clinical application. (**A**) Analysis steps need to be performed for pathogen identification and resistance markers search in clinical samples. (**B**) Existing pipelines and steps they include. Yellow—Illumina data only; green—ONT data only. Connected dots represent steps that are automatically performed in the workflow, whereas isolated dots represent independent analysis steps that need to be called by the user. The figure was created using draw.io.

Several steps should be taken to obtain reliable information on pathogen identity and genotypes from raw sequencing data (Figure 1A). Although the analysis flow is relatively independent of the platform used to generate the data, the software used differs. Here, we will discuss these steps regarding Illumina and ONT data analysis.

#### 3.3.1. Base-Calling and Demultiplexing

Both base-calling and demultiplexing are commonly performed automatically right after sequencing. Many different base callers and demultiplexing algorithms have been developed for the Illumina platform [83,84]. Compared with Illumina, fewer algorithms have been developed for the ONT platform. The MinKNOW software used to drive ONT sequencing has an integrated deep neural network base caller known as Guppy [85]. Using ONT, the user might choose between base calling on the flow after the sequencing is finished or saving raw data and base calling later using more powerful computational resources. It is also possible to choose between three options of base-calling in MinKNOW: fast but least accurate (85–92% median read accuracy), highly accurate, or super accurate base calling with a median read accuracy of 90–96% [85]. Demultiplexing is also commonly performed automatically, and the user only needs to provide a list of barcodes or barcode IDs used during the library preparation.

#### 3.3.2. Adapter Removal and Quality Filtering

Adapter removal and quality filtering are essential to achieve a high-quality genome assembly. Adapters can be removed automatically during the base calling and demultiplexing step. However, it is advisable to perform both removal of the remaining adapters and a quality check before proceeding with the downstream analysis. FastQC for short reads [86] or LongQC [87] and pycoQC [88] for long ONT reads scan raw sequencing data, report per base, per sequence quality statistics, and by searching for overrepresented sequences (i.e., adapters). Commonly used tools for Illumina adapter removal and quality filtering are Trimmomatic [89] or TrimGalore [90], which wraps cutadapt [90] for adapter removal, and FastQC [86] for quality filtering. For ONT sequencing, adapter removal can be performed using Guppy or porechop [91], although the latter has been discontinued and is no longer developed for new library prep versions.

#### 3.3.3. Filtering of Human Reads

This step is essential for routine clinic lab use to protect patients’ identities and privacy. When pathogens are initially isolated from the clinical sample before sequencing, this step can be omitted since no patient-identifiable data will be sequenced along with the pathogen. If, however, a clinical specimen is sequenced as is (for example, the sample directly or from the culture of pus/blood/urine, etc.), removing all human DNA from the dataset is of utmost importance before the analysis starts. Many algorithms can be used for that matter (bbmap, bowtie2, and hisat2), and all of them are based on mapping query data to a reference human genome and removing those reads that map to the reference.

#### 3.3.4. Pathogen Identification

Pathogen identification can be performed both before and after the genome assembly. Since genome assembly is a time-consuming step, we will shortly review the methods to perform assembly-free taxonomy identification. Since Illumina reads are short, only tools that split reads into small ‘words’ (kmers) and compare their mapping to prebuilt index databases can be used. Kraken [92], or its newer version Kraken2 [93], and Centrifuge [94] are commonly used to classify rapidly reads as viral, bacterial, and archaeal domains using the kmer-based approach. On the other hand, ONT reads are long, and in addition to Kraken2 or Centrifuge, a common BLAST-based search against NCBI reference databases (RefSeq/RefProk) can also be used for pathogen ID. We have shown that BLAST provides more accurate results than other tools, with negligible difference in the time taken for the prediction [67].

#### 3.3.5. Genome Assembly and Assembly Quality Control

For Illumina data, the SPAdes [95] assembler is widely used. It contains additional options of metagenome assembly, plasmid assembly, or hybrid assembly, given that long reads are also available for the pathogen. In addition, SPAdes also include an option for additional read error correction before assembly. For ONT data, common assemblers include Canu [96], Minimap/Miniasm [97], Flye [98], and Raven [99]. The Trycycler tool enables a consensus based on results from different assemblers, thus improving the accuracy of the assembly [100]. Our recent publication showed that hybrid assembly constructed using both short and long reads considerably improved plasmid assembly, antimicrobial resistance genes, and identification of virulence factors in clinical isolates [101]. QUAST [102] is commonly used to assess the quality of the assembly by the length of the assembly, the number of contigs, and assembly contiguity. Completeness of the genome assembly can be evaluated using BUSCO [103] or CheckM [104]. GenomeQC comprises both genome assembly statistics and genome completeness assessment [105].

#### 3.3.6. Resistome Identification and Phenotype Inference

Several databases and algorithms are available, and their choice often depends on users’ preferences. Commonly used tools for resistome search include ABRicate [106], AMRFinderPlus [107], ResFinder [108], RGI [109], and Ariba [110]. Ariba and ResFinder can be used on unassembled Illumina data, whereas RGI (CARD), ABRicate, and AMRFinderPlus require assembled contigs/long reads. ONT unassembled reads allow users to identify whether a given antibiotic gene class (such as SHV or CTX-M) is present. However, subtyping the gene is not feasible until genome assembly is performed [67,77]. ResFinder and RGI report antibiotic resistance genes (ARGs) and single mutations associated with antimicrobial resistance and predict a phenotype based on the findings. Choosing the appropriate database is challenging, and our previous publication showed that resistome analysis results could be database dependent [111].

Based on the descriptions of the steps above, the whole sequencing data analysis is challenging for lab personnel unfamiliar with bioinformatic tools. Moreover, most of these tools are command-line based, which also constrain the inclusion of WGS into routine clinical lab use. Therefore, there is an unmet need for automatic bioinformatic pipelines tailored for lab personnel with no bioinformatics and/or command-line use background. Both Illumina and ONT offer in-built user-friendly solutions for taxonomy identification and antibiotic gene search: specific workflows in EPI2ME (ONT) or BaseSpace (Illumina, San Diego, CA, USA) were designed for these tasks. Several open-source pipelines for pathogen identification and AMR surveillance are available such as Bactopia [112], TORMES [113], Nullarbor [114], and CRuMPIT [81] (Figure 1B). However, these workflows are more suited for clinical epidemiology rather than diagnostics. They can hardly be streamlined for routine use in clinics due to a lack of important steps specific to clinics. Moreover, most of them are tailored for Illumina sequence data which is impossible to use for rapid pathogen typing in clinics. To our knowledge, no ONT sequence data analysis automated pipeline, which would include both taxonomy and ARG identification, has been developed to date.

## 4. Machine Learning in Data Analysis for Tackling Antibiotic Resistance

WGS data were mainly used to discern isolates’ genotypes and the presence/absence of antibiotic-resistance genes [115]. In some cases, where antibiotic resistance is highly correlated with ARGs only, this approach showed high concordance between genotype and phenotype. For example, the detection of resistance genes in *Campylobacter jejuni* isolates had a 98% correlation to their phenotype against ciprofloxacin, nalidixic acid, erythromycin, gentamycin, streptomycin, and tetracycline [116]. However, such a high correlation is rare, and in many cases, the detection of ARGs does not associate with the resistant phenotype [117]. Bacteria may become antibiotic-resistant by changing expression levels of porins, efflux pumps, or enhancing biofilm formation [118,119,120]. These changes are impossible to account for using only acquired ARG screening. Machine learning has gained more interest in clinical applications since the rise and availability of next-generation sequencing technologies coupled with advanced and computationally powerful computers.

Machine learning (ML) is a broad set of algorithms, and statistical models used to find connections and correlations between complex data [121]. Its main characteristic that distinguishes ML from other statistical approaches is that the computer learns from the data itself, searching for the peculiarities, i.e., features, in the dataset without being explicitly programmed which features and in which combinations to look for. This approach enables researchers and programmers to elucidate connections in large datasets that are sometimes impossible to find using other conventional statistical models. ML comprises various clustering methods, regressions, decision trees, neural networks, and vector machines, etc. [122].

ML can also aid empirical antibiotic therapy when no AST data for the isolate are available. Feretzakis et al. applied several ML algorithms, including random forest classification, instant-based learning, sequential minimal optimization, and others, to make a weighted empirical decision on which antibiotic should be used based solely on the infectious agent’s Gram stain, the site of infection, and the patient demographics [123]. Although their clinical data were limited to only these three variables, they demonstrated a receiver operating characteristic (ROC) area of up to 0.72 for predicting resistant infections and potentially aiding clinicians to make a more informed decision on antibiotic prescription before time-consuming AST results are in place.

In addition, ML algorithms have been combined with biochemical screening to identify antibiotic targets to enhance drug efficacy [124]. This approach enabled the authors to detect that purine biosynthesis and adenine limitation might increase antibiotic lethality-novel mechanisms that remained undiscovered using only a conventional bioinformatics approach. In 2021, Truong et al. implemented machine learning to enable real-time industrial monitoring of resistance changes in bacterial growth. They measured near-infrared spectroscopy spectra from *E. coli* isolates cultured in 96-well microtiter plates with varying concentrations of tetracycline. They then used partial least squares discriminant analysis (PLS-DA) for discriminating resistant and susceptible strains.

WGS and ML technologies have also been broadly used for improved pathogen identification [125], healthcare outbreak detection [126], elucidating drug effectiveness with regards to microbiome interactions [127], the search for antibiotic mechanisms of action [124], and AST phenotype prediction [26,125,128,129,130]. There are several publications where machine learning enables the prediction of the resistance phenotype based on the genotype [130,131,132]. Some of these models require genome assemblies [128], whereas others use raw sequencing data and thus save the time needed for assembly [130]. Most models yield categorical predictions, i.e., whether the pathogen is susceptible or resistant to the given drug. The Nguyen et al., 2018 [128] models are one of the few that predict the MIC value. However, all phenotype prediction models are heavily skewed towards the dataset used for training. Typically, when these models are used to predict the phenotype of pathogens with a distinct resistance profile, they perform poorly. Due to the relatively low number of isolates with WGS and phenotypic data spanning all possible resistance profiles, the development of the ‘universal’ model seems far-fetched at the time of publication. However, there is a possibility of combining already developed models into so-called ensembles, where data from each isolate are analyzed by all models included, and an isolate is deemed resistant if any of the models predict this class. We have previously used this approach by combining Nguyen et al., 2018 models with AMR-Diag neural networks. We demonstrated that this approach increased prediction accuracy from 25% to 96% for combined data from two datasets [130]. However, most genotype-to-phenotype models have focused on strains isolated from monoculture infections (*E. coli*, *Klebsiella pneumoniae*, and *Acinetobacter baumannii*), and there is a need for the development of an ML-based method for AST prediction of several other antibiotic-bacteria combinations.

## 5. Future Perspectives

**Figure 2:**
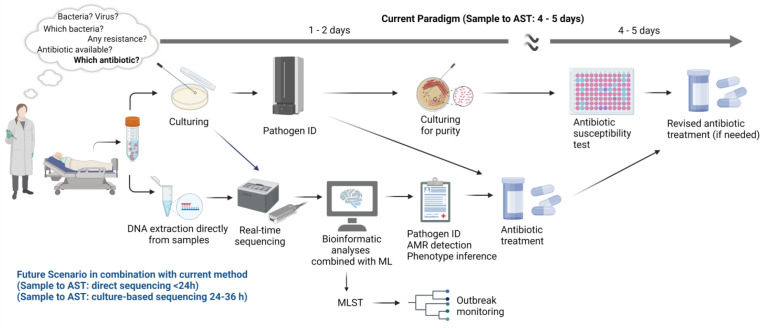
An overview of the current paradigm and future scenario in clinical microbiology. The current paradigm for the diagnosis in the case of infection suffers from a high turnaround time for both pathogen identification and antibiotic susceptibility testing (4–5 days). The potential future scenario with the use of whole genome sequencing has the potential to remarkably reduce the turnaround time. In the future scenario, sequencing can be implemented following direct sequencing from biological material (<24 h) or from bacterial colonies in the culture (24–36 h). Sequencing needs to be followed by proper bioinformatic analyses and ideally combined with a machine learning approach. Created with BioRender.com.

The European Committee on Antimicrobial Susceptibility Testing (EUCAST) and the European Centre for Disease Prevention and Control have indicated that WGS-based detection represents the future of AMR surveillance [133,134]. Rapid pathogen identification, AMR detection, and antibiotic susceptibility testing (AST) are in great demand due to the rise of drug-resistant bacteria. The current paradigm of culture-based AST methods suffers from a long turnaround time (4–5 days) (Figure 2) [67,135]. Genome sequencing seems promising, especially for critically ill patients and in cases where time is critical. In a clinical setting, a combination of direct sequencing from biological material (high bacterial load infections, e.g., a urinary tract infection, mastitis, or meningitis) or bacterial colonies in positive cultures (low bacterial load infection, e.g., bloodstream infection) has the potential to become a routine tool for clinical microbiology (Figure 2). Recent publications show that the use of WGS for pathogen identification and resistance detection has been more widely applied (Table 1). Evidence of their use has increased for some biological samples and bacterial classes. Current sequencing methods involve complex workflows with slow turnaround times and relatively high costs. However, in the recent past, sequencing has become more affordable and more widely applied. However, there is a need for different bioinformatics tools to analyze WGS data, which should adhere to a set of minimum standards and be standardized to be equivalent in terms of the results generated [133]. Such standardization must be implemented at different levels, including the amount of data generated specifically when it comes to third-generation sequencing using different chemistry kits, data quality assessment criteria, cut-off values for trimming and filtering in different software, BLAST cut-off values, taxonomy criteria, and coverage and identity cutoff values for AMR and plasmid detection. In addition, there is a need for reference AMR database curation. Moreover, the bioinformatic tools available today should be assembled into pipelines tailored for use with sensitive data and a user-friendly interface. This allows existing clinical microbiology lab personnel to effectively analyze sequencing data without extensive training in command-line programming.

Interestingly, recent research has demonstrated the potential of the photonics technologies (Raman spectroscopy and phase microscopy) assay as a rapid and first-stage tool for species-, resistance-, and strain-level classification, which WGS can follow up for confirmation [136,137]. This will limit the search space for the sequence alignments so that computations can be performed within seconds. Such a workflow can lead to potential future use in clinical microbiology. Furthermore, recent studies applied Raman spectroscopy to study bacterial composition in dental plaque biofilms [138] and in dry surface biofilms in a hospital environment [139].

The available evidence for using WGS as a tool to infer AST accurately is either poor or non-existent [133]. However, as highlighted by the World Health Organization (WHO) and the EUCAST, there are several major bottlenecks, such as data collection, resistance database establishment, bioinformatic analyses, and establishing a reproducible analytical pipeline [133,140]. Moreover, assessing genotypic data against phenotypic data (i.e., clinical breakpoints) represents a more formidable challenge. This will be necessary if WGS-based testing is to guide clinical decision-making. However, there is an urgent need for agreement on the appropriate and effective principles and quality control metrics to facilitate standardization and harmonization of analytical approaches and interpretative criteria for WGS-based predictive AST. More focused studies and resources are needed as a priority to improve knowledge. The ML AST tools should also be developed considering the requirements for artificial intelligence robustness set forth by the European Commission [141,142]. The developers of these tools should thoroughly assess whether the ML system meets all the standards, not only for robustness and accuracy but also vulnerability against tampering, biases caused by uneven sampling, and compliance with data confidentiality.

## Figures and Tables

**Table 1:** The use of real-time metagenomic Oxford Nanopore sequencing (mONS) for infection identification and ARG detection. LRTI: lower respiratory tract infection, UTI: urinary tract infection, BSI: bloodstream infection, SSI: surgical site infection, PJI: prosthetic joint infections, IBS: intraoperative bile aspirates, JF: joint fluid, PT: prosthetic tissue. * NR: not reported, ** the number of analyzed samples <10, and sensitivity/specificity estimates should be treated with care.

Disease	Sample Type	Number of samples	Culture Independent	Turnaround Time (h)	Sensitivity (%)	Specificity (%)	ARG Detection	Main Outcome	Reference
LRTI	Sputum	81	yes	6	96.6	41.7	yes	mONS can rapidly and accurately characterize bacterial LRIs and might contribute to a reduction in broad-spectrum antibiotic use	[70]
UTI	Urine	86	yes	NR *	71–100	-	yes	mONS pathogen and resistome detection for the diagnosis of UTIs warrants prospective clinical validation	[71]
		76	yes	6	86.7	96.8	yes	mONS is a promising clinical diagnostic tool for infectious diseases	[72]
Meningitis	Cerebral spinal fluid	1	yes	~5	-	-	yes	mONS enabled the direct diagnosis of meningitis caused by *Pasteurella multocida*, which is not routinely detected by current point-of-care assays	[73]
		1	yes	2	-	-	yes	mONS enabled the diagnosis of community-acquired Streptococcus pneumoniae meningitis, providing a less than two-hour workflow including only a 20 min sequencing time, detection, identification, typing, and an in silico antibiogram	[74]
		1	yes	6	-	-	yes	mONS could be considered for the point-of-care diagnosis of infectious meningitis, by direct identification of pathogenic genomes and their genotypes/serotypes	[75]
		285	yes	NR	100	94.4	no	16S rDNA mONS was more effective than conventional culture in postoperative bacterial meningitis and may contribute to evidence-based decisions for antibiotic maintenance and discontinuation	[76]
BSI	Blood	8	no	3.5–6	100 **	83.1 **	yes	Identification of pathogens was possible after 10 min of sequencing and all predefined AMR-encoding genes and plasmids from monoculture experiments were detected within one hour using raw mONS data	[67]
		2	no	6.5	100 **	-	yes	Flongle data allowed for rapid bacterial ID and resistome detection based on the first 1000–3000 generated sequences (10 min to 3 h from the sequencing start)	[77]
SSI	IBS	42	yes	6–14	100	-	yes	The results generated using mONS were similar to the current methods of detection but were obtained in a significantly shorter amount of time. mONS could be used to tailor antibiotics in surgical patients and reduce the use of broad-spectrum antibiotics	[78]
		42	yes	8	100	100	yes	Rapid microbial profiling with mONS is feasible with broader organism and resistance profiling compared to standard cultures. mONS has perfect negative predictive values and can potentially improve antibiotic stewardship	[79]
Mastitis	Milk	24	yes	9	100	92.3	yes	A proof-of-concept study that established an effective method for host removal and direct MinION sequencing from mastitis milk	[80]
PJI	Sonication fluids	7	yes	NR	100 **	100 **	no	A novel, scalable pipeline for the real-time analysis of MinION sequence data and the use of this pipeline to show initial the proof of concept that mONS can provide rapid and accurate diagnosis for prosthetic joint infections	[81]
	JF and PT	9	yes	14–22	100 **	80 **	yes	A proof-of-concept study that established that mONS can function as a rapid and accurate tool in PJI diagnostic microbiology	[82]
